# Specifics of socialization of children with autism spectrum disorders

**DOI:** 10.1192/j.eurpsy.2024.1368

**Published:** 2024-08-27

**Authors:** A. Akhmetzyanova, T. Artemyeva, A. Minullina

**Affiliations:** Department of Psychology and Pedagogy of Special Education, Kazan Federal University, Kazan, Russian Federation

## Abstract

**Introduction:**

One of the important tasks of modern education is the adaptation of children with autism spectrum disorders to the social space, which allows them to ensure their personal development and self-realization.

**Objectives:**

Study of the specifics of the socialization of children of preschool age with autism spectrum disorders.

**Methods:**

The study involved 27 preschool children with autism spectrum disorders attending an educational institution for children with disabilities; 6 were girls and 21 were boys; 20 children with intact speech and 7 children with speech disorders. The following methods were used: “Map of manifestations of activity by A.M. Shchetinina, N.A. Abramova; “Map of observations of the manifestations of communicative abilities in preschool children” A.M. Shchetinina, M.A. Nikiforova; “Emotional faces” N.Y. Semago.

**Results:**

It was found that children with autism spectrum disorders have the greatest severity of such activity indicators as “is in a good mood” (1.67), “shows stubbornness” (1.56) and “shows great mobility” (1.56). Among the manifestations of communicative abilities in preschool children, the most developed parameter is: “sincere in his statements, in the manifestation of his feelings” (2,07). At the same time, the lowest expression of communication skills (0.96) in children with autism spectrum disorders is observed in terms of: “has organizational skills”, “the child seeks to understand the other, his thoughts, feelings”; “observant, sees and realizes the characteristics of other children and adults”. The least pronounced indicator is observed in the indicator of initiative; children do not show initiative in communication, have difficulty understanding and supporting the initiative of another child in an interaction situation. Children have a low level of operational communicative actions and skills: children are not expressive in communication, do not master verbal means of communication and are not able to maintain contact with communication partners.

**Conclusions:**

The results obtained in the study confirm the need to develop and implement psychological and pedagogical programs aimed at developing social skills in preschool children with autism spectrum disorders. This paper has been supported by the Kazan Federal University Strategic Academic Leadership Program.

**Disclosure of Interest:**

None Declared

